# Challenges and opportunities for China entering global research and development for emerging infectious diseases: a case study from Ebola experience

**DOI:** 10.1186/s40249-020-00643-0

**Published:** 2020-03-13

**Authors:** Chao Li, Jing-Yi Chen, Yang-Mu Huang

**Affiliations:** 1 Public Health Emergency Center, Chinese Center for Disease Control and Prevention, Beijing, China; 2 School of Public Health, Peking University, 38 Xueyuan Road, Haidian District, Beijing, 100191 China; 3 Harvard T. H Chan School of Public Health, Boston, USA

**Keywords:** Emerging infectious disease, Research and development, Ebola virus disease

## Abstract

**Background:**

China has emerged as a powerful platform for global pharmaceutical research and development (R&D) amid the 2014 Ebola outbreak. The research and development impact of developing countries on prevention and control of infectious disease outbreaks has long been underestimated, particularly for emerging economies like China. Here, we studied its research and development progress and government support in response to Ebola outbreak by timeline, input, and output at each research and development stage. This study will contribute to a deeper understanding of the research and development gaps and challenges faced by China, as well as providing evidence-based suggestions on how to accelerate the drug development process to meet urgent needs during future outbreaks.

**Methods:**

Data were obtained from the National Nature Science Foundation of China database, PubMed database, Patent Search System of the State Intellectual Property Office of China, National Medical Products Administration, national policy reports and literature between Jan 1st, 2006 and Dec 31st, 2017. An overview of research funding, research output, pharmaceutical product patent, and product licensed was described and analyzed by Microsoft Excel. A descriptive analysis with a visualization of plotting charts and graphs was conducted by reporting the mean ± standard deviation.

**Results:**

China has successfully completed the research and development of the Ebola Ad5-EBOV vaccine within 26 months, while the preparation and implementation of clinical trials took relative long time. The National Nature Science Foundation of China funded CNY 44.05 million (USD 6.27 million) for Ebola-related researches and committed strongly to the phase of basic research (87.8%). A proliferation of literature arose between 2014 and 2015, with a 1.7-fold increase in drug research and a 2.5-fold increase in diagnostic research within 1 year. Three years on from the Ebola outbreak, six Ebola-related products in China were approved by the National Medical Products Administration.

**Conclusions:**

China has started to emphasize the importance of medical product innovation as one of the solutions for tackling emerging infectious diseases. Continuing research on the development of regulatory and market incentives, as well as a multilateral collaboration mechanism that unifies cross-channel supports, would advance the process for China to enter global R&D market more effectively.

## Background

The unprecedented outbreak of Ebola virus disease (EVD) in 2014–2016 caused a total of 9216 reported cases and 4555 deaths in African countries [1]. The widespread virus featured by the high mortality rate triggered a collective international response to halt the ongoing transmission [2, 3]. The international community launched multiple actions and invested millions of dollars in scaling up efforts, which were unprecedented for a disease outbreak [4, 5]*.* Facing the situation of no available vaccine and only limited ongoing drugs and diagnostics researches before the outbreak, supports for research and development (R&D) of relevant medical products were crucial not only for disease control and prevention, but also for reducing the social-economic threats from this fatal virus.

The outbreak triggered a rapid scale-up global response, including R&D efforts supported by governments and private sectors [6]. Since then, the R&D for controlling the epidemic entered the speedway. After a collaborative and inclusive effort of regulators, scientists, and manufacturers, several medical products had been developed and demonstrated promising. Drug candidates such as ZMapp and TKM-Ebola showed antiviral efficacy in vitro and vivo, and were determined by the World Health Organization (WHO) for an emergency or compassionate use [7, 8]. In August 2019, another drug candidate REGN-EB3 was tested in a randomized, controlled trial, and delivered a low mortality rate of around 6.0% [9]. The rVSV-ZEBOV vaccine was proven to be highly effective after its phase 3 trial [7, 10], and had already been deployed to the Democratic Republic of Congo during the 2018 Ebola outbreak under a compassionate use of the WHO’s Expanded Access Framework [11]. The rVSV-ZEBOV vaccine yielded highly efficacious and protective results among high-risk contacts based on the fact that only 8.8% of the vaccinated population reported Ebola cases, and only 2.2% cases were reported 10 days or more after vaccination [12].

The scale of the West African outbreak promoted China, for the first time, to implement a series of actions for combating this fatal emerging infectious disease (EID) [13]. Besides providing personnel and material assistance to the epidemic areas, China has facilitated the development of diagnostic reagents, therapeutic drugs and vaccines for the Ebola virus. A restructured Ebola vaccine independently developed by China was approved by the former China Food and Drug Administration (CFDA) in November 2017 under the emergency use. China has become the third country with a developed vaccine to combat EVD after the United States and Russia. This significant breakthrough in Ebola-related R&D paves a smoother path for China to engage in the prevention and control of global EIDs.

As one of the largest developing and emerging countries, China was expected to contribute more on global response towards EIDs. The unprecedented investment China has made in developing Ebola-related products, not only strengthened its national strategies to protect the large population, but also stressed China’s strong willingness to participate in global health governance and R&D market. With consistent inputs to R&D sectors, more competitive products and technologies would be generated with profitable economic returns.

Despite the rapid R&D responses given to Ebola outbreak, far too little attention has been paid on exploring China’s existing R&D opportunities and challenges. Some studies have introduced China’s efforts on the development of certain drug and vaccine candidates for Ebola [14]. Others focused on its contribution to implementing prevention programs such as mobile laboratory [15]. However, previous published articles were limited to analyzing the Ebola-related R&D processes and funding flow in China [16]. This research aims to illustrate China’s current situation and government support in implementing R&D response for Ebola by studying the timeline, input and output at each R&D stage. It will contribute to a deeper understanding of the R&D gaps and challenges faced by China, as well as providing evidence-based suggestions on how to accelerate the drug development process to meet urgent needs during future outbreaks. Moreover, this research could provide peer experience for other developing countries on promoting relevant R&D more effectively, which could contribute eventually to global EID prevention and control.

## Methods

### Data source and data description

Data were extracted from the National Natural Science Foundation of China (NSFC, http://www.nsfc.gov.cn), the State Intellectual Property Office of China, the National Medical Products Administration (the former CFDA), as well as national policy reports and literature, between 1 January 2006 and 31 December 2017.

#### The Chinese government’s R&D expenditures

Chinese government’s R&D expenditure data was collected from the NSFC website, which was one of the main public channels to support scientific research [17]. Search terms included “Ebola” and “Ebola virus” in both Chinese and English languages. Exclusion criteria included duplicated researches, researches on Ebola epidemic studies, emergency information platform studies, and other topics unrelated to Ebola R&D process. Since NSFC only supports pre-clinical research, the included projects were divided into “Basic research” and “Development” according to the abstract. “Basic research” was then divided into “Etiology research” and “Pathogenesis research”. “Etiology research” were studies that examine the cause and origin of the disease. In this case, etiology research mainly focused on the structure and the genome of the Ebola virus. “Pathogenesis research” mainly emphasized the biological mechanism progress of the disease. To analyze the input status by each project category, “Development” was divided into “Vaccine pre-clinical development”, “Diagnosis pre-clinical development” and “Drug pre-clinical development”. For the projects without “Ebola” in the topics, the Ebola-related studies were also included as subprojects. The method was summarized in Table 1. These projects were defined as “Ebola subprojects” and the data were also collected using “Ebola” and “Ebola virus” terms. All the results were included by the relevance of topic after scanning the project introduction.

**Table 1:** Method for categorizing Chinese government’s research and development expenditures

Catogory	Sub-catogory	Description
Basic	Etiology research	Studies examined the casue and origin of the disease, mainly focused on the structure and the genome of the Ebola virus
	Pathogenesis research	Studies examined biological mechanism progress of the disease
Development	Vaccine pre-clinical development	Studies examined Ebola related vaccine pre-clinical development progress
	Diagnosis pre-clinical development	Studies examined Ebola related Diagnosis pre-clinical development progress
	Drug pre-clinical development	Studies examined Ebola related Drug pre-clinical development progress

#### Ebola R&D-related English scientific research output

We used GoPubMed as the search tool to conduct bibliometric analysis for literature from 1 January 2006 to 31 December 2016. GoPubMed is a web search tool that enables extracting terms from the retrieved abstracts, and presenting the induced ontology by the input of keywords to PubMed [18]. English scientific papers were searched using “Ebola virus” as the MeSH term in Gopubmed and filtered under the category “Chemical and drugs”, “Vaccination” and “Diagnostic”. Articles written in Chinese were searched by the Wanfang database (http://www.wanfangdata.com). the search terms were Chinese using (“Ebola”)*(Topic:(Research and Development) + Topic:(Vaccine) + Topic:(Antibody) + Topic:(drug) + Topic:(diagnosis). Depulicated and unrelated literature were excluded in accordance to the same criteria for projects. These papers were then classified into three categories of “Chemistry and medicine”, “Vaccination” and “Diagnostics”, by their topics and abstracts. Full articles were obtained for further exclusion if needed.

#### China Ebola pharmaceutical products patent

China Ebola Pharmaceutical Products Patent data were extracted from the Patent Search System of the State Intellectual Property Office of China (http://www.sipo.gov.cn). We used both Chinese and English “Ebola” and “Ebola virus” as the search terms, and divided them into “Drugs”, “Vaccines” and “Diagnostics” through the skimming of patent descriptions. At the same time, the number of patent applications and information about patent authorizations were collected for analysis.

#### Product approval

Product approval data were extracted from the website of the National Medical Products Administration (the former CFDA, http://english.nmpa.gov.cn). We used both Chinese and English “Ebola” and “Ebola virus” as the search terms in the “National drug” and “Medical instruments and equipment” databases, and classified them into the category of “Drugs”, “Vaccines” and “Diagnostics”.

#### Timeline of China Ebola R&D

The indicators were the key time points for the development of major Ebola pharmaceutical products, including clinical trial approval time, clinical trial period, and approval time for products available for the market. Data were mainly obtained through relevant announcements and articles issued by the Chinese agencies.

## Data analysis

All the data were double-entered into Microsoft Excel (Version16.34, Product ID: 02954–060-367 900, Seattle, the United States) and checked for consistency. Microsoft Excel was used for analysis and description. Microsoft Excel was also used for data analysis and description. We extracted data from the data source and conducted a descriptive analysis by reporting the mean ± standard deviation as well as virtualized the data by plotting charts and graphs. There is no statistical testing involved in the analysis. The funding value for R&D was reported in USD, calculated according to the exchange rate in that year.

## Results

### The Chinese government’s R&D expenditures

A total of 14 projects and CNY 44.05 million (USD 6.27 million) were invested in Ebola researches through NSFC. Funding for basic research (87.7%) was much higher than that for early development (12.3%). Among all the basic projects, CNY 7.57 million (USD 1.08 million) (19.6%) was invested for etiological studies, and CNY 31.07 million (USD 4.42 million) (80.4%) was for pathogenic mechanism studies. The total investment for Ebola research subprojects was CNY 21.74 million (USD 3.10 million), among which 75.2% was on the pathogenic mechanism. Only 1.0 and 23.8% of the total investment were on etiological research and preclinical development projects, respectively.

**Fig. 1:**
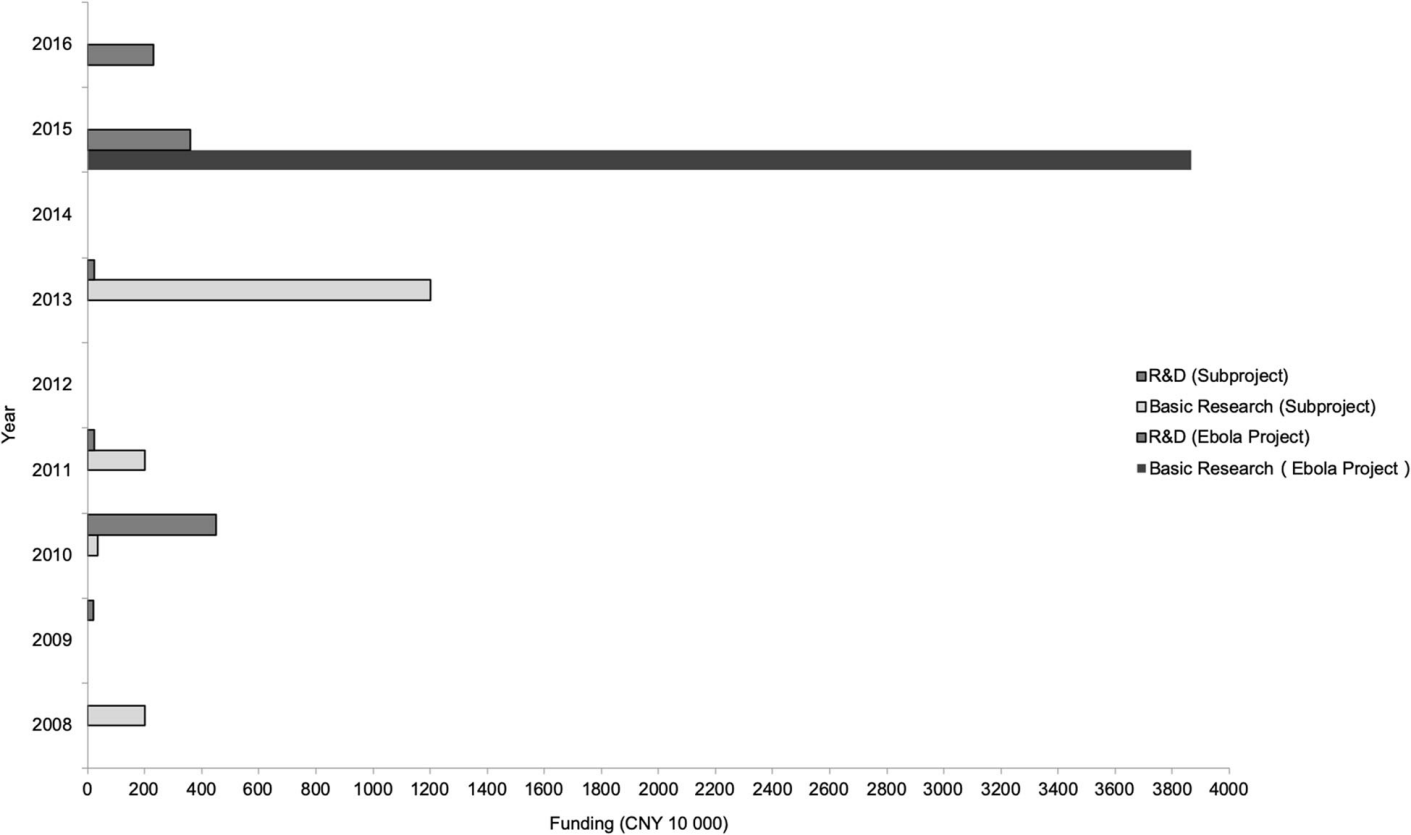
Funding approved for Ebola research projects by National Natural Science Foundation of China

Before 2014, there was no recorded project approved for Ebola. However, some Ebola basic research projects were approved as subprojects that embedded under projects such as “viral pathogen research” or “specialty drug research”. After 2014, the number of projects under the “basic research” and “preclinical development research” categories have undergone tremendous growth. Basic research projects received CNY 38 million (USD 5.40 million) and drug R&D researches received CNY 3.5 million (USD 0.50 million) in the year of 2015. Projects that included Ebola subprojects were approved mostly in 2008, 2010, 2011 and 2013. No Ebola projects after 2014 were approved as independent projects (Fig. 1).

### Ebola R&D related research outputs

**Fig. 2:**
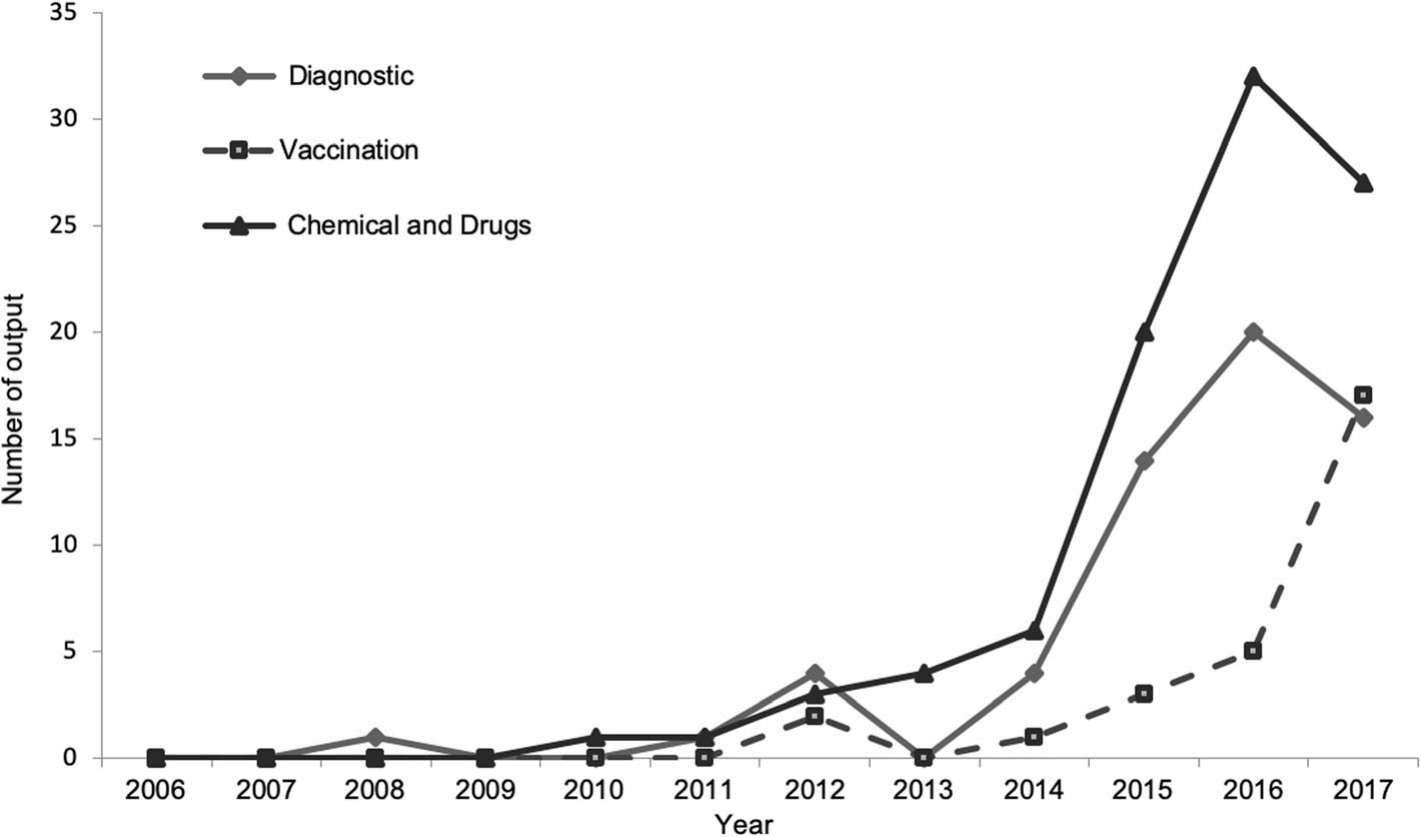
Scientific outputs of China developed Ebola medical products

A total of 182 English scientific articles related to Ebola vaccines, drugs, and diagnostic were published between 2006 and 2017 (Fig. 2) by Chinese researchers. Most studies focused on drug (94 articles) and diagnostics studies (60 articles), while only 28 articles were vaccine-related. Ebola-related researches rose from 11 associated studies in 2014 to 60 studies in 2017. The number of Ebola vaccine-related articles peaked in 2017 with a 2.4-fold increase compared to that in 2016. From 2014 to 2016, the number of drug and diagnostic researches has also increased. Notably, a proliferation of literature arose between 2014 and 2015, with a 1.7-fold increase in drug research and a 2.5-fold increase in diagnostic research annually.

**Fig. 3:**
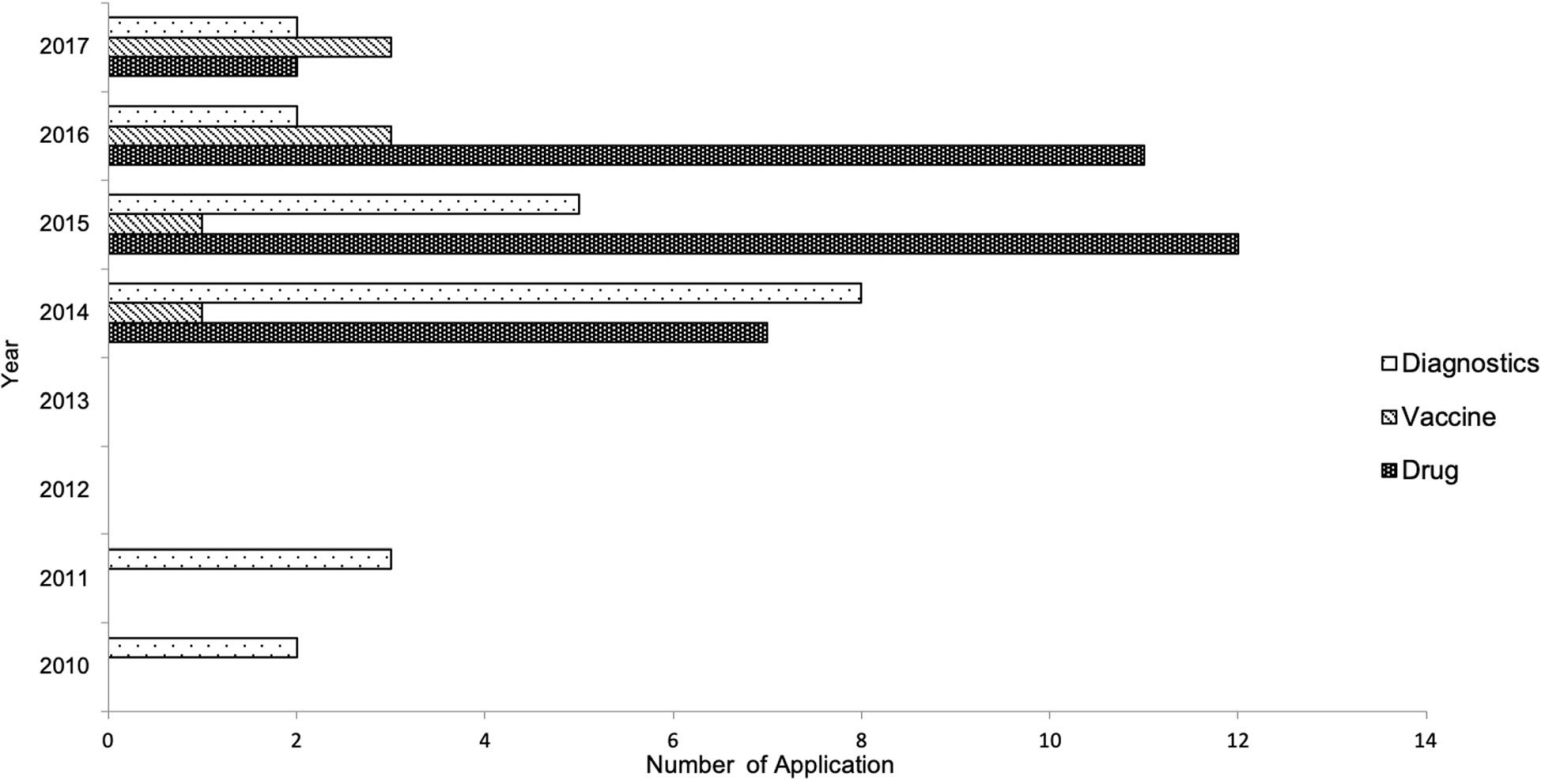
Patent application of China developed Ebola medical products

Similarly, patent application for China’s Ebola products has increased sharply since 2014 (Fig. 3). Before the disease outbreak, there were no patent application for vaccines or drug products. However, the number of drug products increased by 71.4% between 2014 and 2015. The vaccine and diagnostic products increased by 200.0 and 240.0%, respectively, between 2014 and 2016. By 2015, 18 patents for Ebola drug products have been approved in China, including 5 for diagnostic reagents, 1 for a vaccine, and 12 for drugs.

Up to 2018, six Ebola-related products have been approved by the National Medical Products Administration, including one vaccine product and five diagnostic products (Table 2). Among them, 75.0% approved products were applied by privately owned enterprises.

**Table 2:** Main manufacturers of China Ebola approved products

Category	Product	Manufacturer	Type of manufacturer	Approved time
Vaccine	Recombinant Ebola virus* disease vaccine (Adenovirus type 5 vector)	Cansinotech	Privately owned enterprises	16 November 2017
Diagnostics	Ebolavirus RT-PCR kit	BGI-Tech	Privately owned enterprises	16 February 2015
Diagnostics	Ebolavirus RT-PCR kit	Tianlong Technology	Privately owned enterprises	28 January 2015
Diagnostics	Ebolavirus RT-PCR kit	Puruikang Biotechnology	Privately owned enterprises	25 November 2014
Diagnostics	Ebolavirus RT-PCR kit	Daan Gene	state-owned enterprises	25 November 2014
Diagnostics	Ebolavirus RT-PCR kit	Shanghai ZJ BioTech	Privately owned enterprises	25 November 2014

* Approved by the State Food and Drug Administration of China only under emergency use

### R&D timeline for Ebola-related medical products

**Fig. 4:**
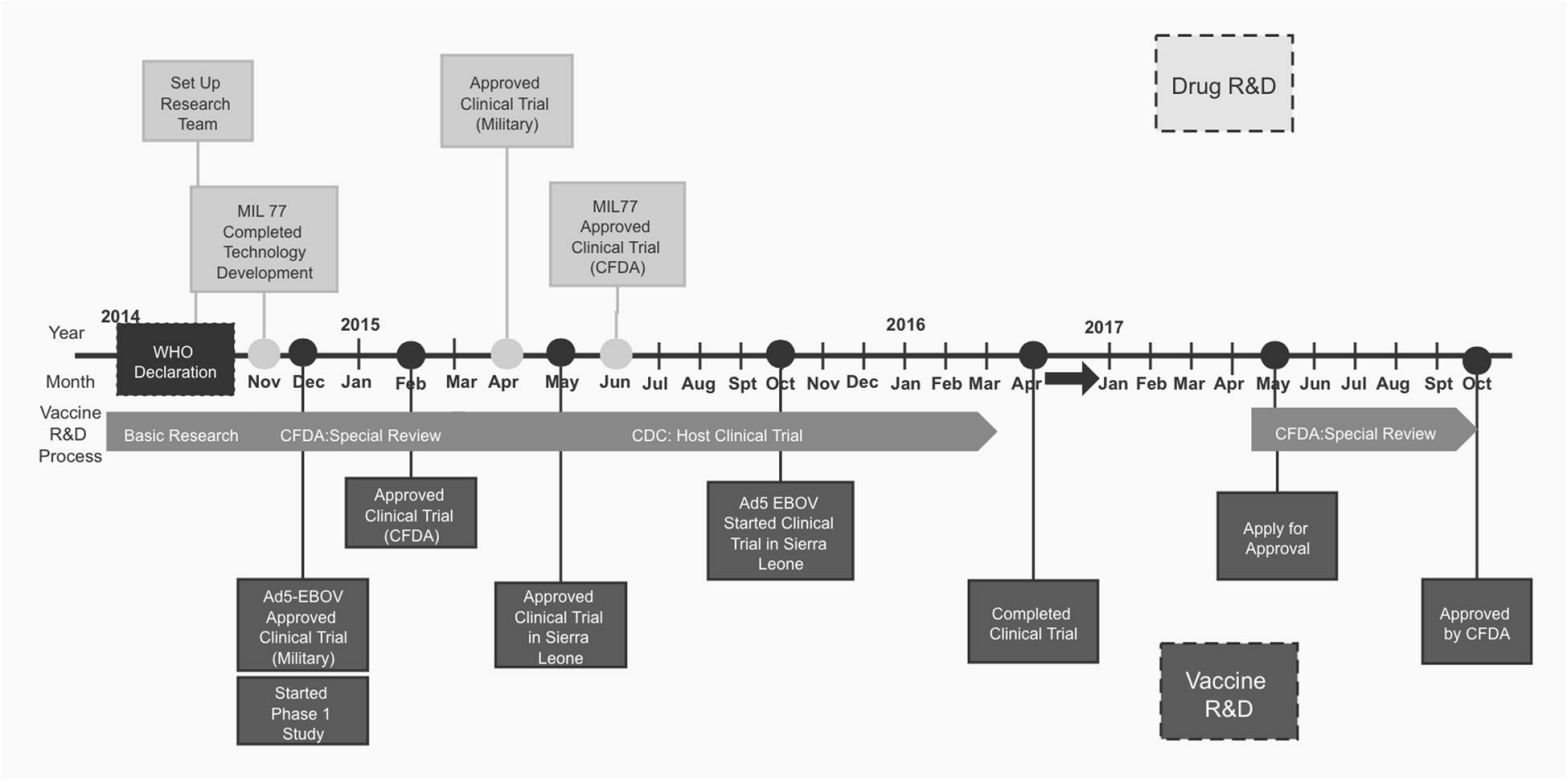
Timeline of research and development Process of China developed Ebola vaccine and drugs. *R&D* Research and development; *CFDA* State Food and Drug Administration of China; *CDC* Centers for Disease Control and Prevention

Under the support of the National High-tech R&D Program (863 programs), China completed the R&D of Ebola Ad5-EBOV vaccine within 26 months. The timeline in Fig. 4 showed that the basic research, supported by the national National High-tech R&D Program of China (863 program), immediately started after the 2014 Ebola outbreak. The military and the former CFDA approved Phase 1 clinical trial study through the special review process. However, China spent 7 months to get the approval for overseas phase 2 clinical trials in Sierra Leone and spent another 6 months for the preparation of the trial [19]. As the Center for Drug Evaluation started to follow up on the vaccine development process in 2014 and initiated the special review process, China’s vaccine market approval ran more efficient with a considerably shortened awaiting time for market approval [20]. In May 2017, the product submitted the application of preventive biological products and got approval in October under the emergency usage. The product was later completed after the process of phase 3 clinical trial [21].

China’s anti-Ebola MIL77 drug was developed by the Institute of Basic Medical Sciences of the Academy of Military Medical Sciences and Beijing Mabworks Biotech Co. Ltd. team. The research team was established in August 2014, and used the drug to treat a British Army nurse who recovered from Ebola at a London hospital in March 2015. Even though the clinical approval document was obtained in April 2015, the clinical trials back then have not begun yet.

## Discussions

As one of the most severe and ongoing EIDs during the past few decades, Ebola has illustrated the high burden of EIDs on global economies and public health, and has brought valuable lessons on how to perform effective actions towards control and prevention for EIDs globally [22, 23]. China’s multiple strategies in response to the Ebola outbreak, including actions on encouraging related R&D processes, revealed its persistent participation and commitment to combating global EIDs [24]. In this study, a descriptive analysis on R&D input and output at pre-clinical, clinical and licensing stages was carried out to identify China’s breakthrough and potential gaps in supporting Ebola-related R&D. It demonstrated the performance of China’s current R&D process and relevant policy supports, in the hope of sharing experiences and major constrains in developing timely and efficient EID products. Our results implicated that future mechanisms and policies to accelerate the R&D process for EIDs need to be established timely by researchers and regulators.

Our study showed that the number of Ebola-related research funding, scientific outputs and patent applications increased tremendously since the 2014 Ebola outbreak. The unprecedented growth illustrated China’s determination and capacity in improving the R&D process for EIDs. The rapid increase of expenditure and investment on drug and vaccine development, as well as the former CFDA’s record-breaking time interval for Ebola vaccine approval, demonstrated China’s swift and sufficient response to the development of Ebola medical products. However, the number of products entering the advanced development phases was quite small compared with the number of basic researches. Based on the performance by each stage, our results suggested that the potential gaps include the lack of EIDs’ pre-outbreak preparation mechanism; fragmented processes and insufficient incentives to translate basic research to advanced development phase efficiently; and the immature public and private partnership (PPP) model adopted for R&D, especially for those that lack of domestic clinical trial capacity.

### Lack of pre-outbreak preparation mechanism

Our results showed that although the number of approved Ebola research projects and funding increased significantly after the 2014 Ebola outbreak, no such project was approved by the NSFC before 2014. Due to NSFC’s unique open-topic application and selective peer-review system, our results indicated inefficient awareness of preparedness for EID related R&D from both the scientists and the authorities. However, R&D for medical products is a time-consuming process, which moves much slower than the pandemic. Thus, performing a risk assessment to identify the potential EID threats and setting priorities for R&D preparedness is essential for the timely response [25–27]. Besides, the results spotted many Ebola-related research outputs were from other irrelevant projects before 2014. This issue might be explained by the limited national support on Ebola before its outbreak and the relatively slow process for new project funding approval. Establishing an emergency funding mechanism is one of the key determinants for accelerating the R&D process.

Moreover, a scientific and advanced national R&D strategy for EIDs should be developed. Experiences from the United States illustrated the importance of setting emergency priority and budget for potential biomedical threats before the outbreaks. US’s National Institute of Allergy and Infectious Disease (NIAID) identifies and assesses its emerging and re-emerging infectious disease portfolio based on a yearly-basis and then determined the required settings that include funding budgets, inventory management, deployment and administration strategies [28]. Since 2004, the NIAID has invested over USD 333 million on Ebola basic research and research toward the development of vaccines, therapeutics, and diagnostics, which led to at least a dozen vaccines and therapeutics in the pipeline for Ebola before the 2014 outbreak [29].

### Low translation from basic studies to advanced development

The flat translation rate shown in this study might be related to a lack of funding and technical support for early development and clinical trials and fragmented coordination between research institutions and private sectors for research translation. Though the government has funded a massive amount on early-stage basic research soon after the Ebola outbreak, limited resources were allocated to the later stages of the innovation process. This could echo the results that only 12.3% of NSFC projects were related to early development, and none for clinical trials. Even though regulators have realized the importance of R&D translation, and launched the “New Drug Development Programme” since 2008 with a total of CNY 128 billion (USD 18.21 billion), only a few projects focused on infectious disease, let alone EIDs [30].

### Immature public and private partnership

The coordination mechanisms between research institutions and pharmaceutical companies are crucial for accelerating the R&D process for EIDs. Multiple studies have demonstrated the importance of public private partnership (PPP) [28, 31]. In China, public research institutions are the main actors for basic research, whereas private companies mainly work on development, candidate product manufacturing and massive production. For example, the successful R&D for the Ebola vaccine in China was developed by China’s Academy of Military Medical Sciences, along with CanSino Biotech. However, PPP is still limitedly seen, especially for developing EID related countermeasures. Reasons might include limited incentives or support for academic institutions to transform research into products, as well as limited market incentives for private industries to invest in product development, especially when the development process requires international coordination. Our study showed that China spent nearly a year to complete the preparation for overseas clinical trials. Though the Chinese government has shown strong support for coordinating this overseas clinical trial, more mature clinical research partnerships should be established, like the Partnership for Research on Ebola Vaccines in Liberia initiated by NIAID [32]. Governmental supports are essential during EID-related R&D processes, especially in supporting overseas clinical trials, strengthening clinical research network, providing clinical training and accelerating the registrations to help survive the “Dead Valley” [33], the period that most of the drugs fail from transforming basic researches to clinical trials.

### Looking forwards: improvements and opportunities

The Ebola outbreak has pushed China forward to be more engaged in global EID responses, including participated in Ebola-related R&D. As discussed, China has taken multiple actions to support the domestic R&D processes, such as funding for basic research, and coordination for the overseas clinical trial. Moreover, the Chinese government has made great efforts to initiate special review regulations for the acceleration of Ebola products’ approval. Comparing with the standard 150 workdays’ time frame in China, the approval for Ebola vaccine only took 83 workdays, setting a new time record for New Drug Application approval at that time. Drug approval regulations might turn out to be a sufficient incentive for products without market returns. For example, the priority review voucher program for neglected tropical diseases has demonstrated valid for policymakers to encourage beneficial investments from industry [34, 35]. Similarly, China’s review and approval system reform was aimed to stimulate industry innovation [36]. The timely approval process for Ebola vaccine reflected China’s successful reforms on drug approval regulations for incentivizing new drug development and improving the efficient usage of medical countermeasures during the emergency. Besides the special review procedures, the government supports and coordination mechanism played an equally important role in accelerating the licensing process for candidate products. For example, the Clinical Department of the Food and Drug Administration could provide technical guidance and assistance during clinical trials, which would enhance the efficiency of data mining and experimental design.

China’s determination to reform on drug approval policies is shaping the evolutional path for drug R&D innovation. China has established the national strategy to spur pharmaceutical industry innovation in order to increase competitiveness of Chinese industries in the global drug market [37, 38]. Moreover, the former CFDA was approved as a new regulatory member of the International Council for Harmonization in June 2017, which enabled China to learn the latest regulatory scientific outcomes and advanced regulatory concepts in the global level. It helped improve the national regulatory to meet the international standards, and provide incentives for private pharmaceutical companies on R&D for infectious diseases.

Moreover, the increasing international community collaboration would provide more opportunities for countries, including China, to better participate in global EID control and prevention. For example, the Coalition for Epidemic Preparedness Innovations (CEPI) launched a coordinated, international, and intergovernmental plan which aimed to develop and deploy new vaccines to prevent future epidemics. It might provide additional supports and incentives for Chinese actors. The funding and technical supports provided by the CEPI could potentially compensate some R&D constrains China is encountering, as mentioned above. China could also take advantage of the CEPI platform technologies to increase its development capacity. Besides, China’s regulators should establish a streamlined regulatory process and a plan for pre-outbreak preparation and drug approval. Last but not least, developing integrated coordination mechanisms for overseas clinical trials and sufficient incentives are crucial for private sectors to be better involved during the R&D process for EIDs.

Although this study represented the most timely information, several limitations still existed. First, we might underestimate the real public funding. Because data that are not publicly available on official websites were not included in this study. Currently, only the funding data from the National Natural Science Foundation of China can be obtained, which mainly focused on basic research. Second, Gopubmed was used as the search tool for bibliometric analysis, which helped classify the types of products of publications according to the article abstracts and keywords. Thus the possibility of misclassification largely depends on the tool’s robustness and accuracy. To overcome the data unavailability, we consulted relevant researchers and regulators to confirm the data collected.

## Conclusions

This study illustrated China’s breakthrough in dealing with Ebola from an R&D perspective, especially in establishing regulatory reform to support and accelerate Ebola vaccine approval. In recent years, China has recognized that the R&D of medical products has become an essential solution for combating global EIDs. At the same time, China has also adopted policies and improved regulations to ensure an efficient R&D process and to encourage pharmaceutical companies to invest in R&D for EIDs. However, China still faces many challenges in the process of transforming basic research into medical products and implementing overseas clinical trials. In the future, China needs to strengthen its government support further and to deepen R&D related system and mechanism reform to cope with future threats from EIDs.

## Data Availability

The datasets used and/or analysed during the current study are available from the corresponding author on reasonable request.

## Abbreviations

CEPI: Coalition for epidemic preparedness innovations
CFDA: China Food and Drug Administration
EID: Emerging infectious disease
EVD: Ebola virus disease
NIAID: National Institute of Allergy and Infectious Disease
NSFC: National Natural Science Foundation of China
PPP: Public private partnership
R&D: Research and development
WHO: World Health Organization
